# Photocatalytic Abatement of Volatile Organic Compounds by TiO_2_ Nanoparticles Doped with Either Phosphorous or Zirconium

**DOI:** 10.3390/ma12132121

**Published:** 2019-07-01

**Authors:** Melodj Dosa, Marco Piumetti, Samir Bensaid, Tahrizi Andana, Camilla Galletti, Debora Fino, Nunzio Russo

**Affiliations:** Department of Applied Science and Technology, Politecnico di Torino, Corso Duca degli Abruzzi 24, 10129 Turin, Italy

**Keywords:** VOCs abatement, TiO_2_-based photocatalysts, photocatalysis, ethylene oxidation, propylene oxidation

## Abstract

The aim of this work is to study the activity of novel TiO_2_-based photocatalysts doped with either phosphorus or zirconium under a UV-Vis source. A set of mesoporous catalysts was prepared by the direct synthesis: TiO_2__A and TiO_2__B (titanium oxide synthesized by two different procedures), P-TiO_2_ and Zr-TiO_2_ (binary oxides with either nonmetal or metal into the TiO_2_ framework). Complementary characterizations (N_2_ physisorption at 77 K, X-ray powder diffraction (XRD), field emission scanning electron microscopy (FESEM), energy dispersive X-ray (EDX) analysis, X-ray Photoelectron Spectroscopy (XPS), and (DR)UV-Vis spectroscopy) were used to investigate the physicochemical properties of the prepared catalysts. Then, the photocatalysts were tested for the oxidation of propylene and ethylene under UV-Vis light. As a result, the most promising catalyst for both the propylene and ethylene oxidation reactions was the P-TiO_2_ (propylene conversion = 27.8% and ethylene conversion = 13%, TOS = 3 h), thus confirming the beneficial effect of P-doping into the TiO_2_ framework on the photocatalytic activity.

## 1. Introduction

Over the last few decades, the abatement of volatile organic compounds (VOCs) has received much interest [1,2,3]. Studies have shown that VOCs are highly responsible for several environmental problems, such as the enforcement of the greenhouse effect, the depletion of stratospheric ozone, the formation of secondary organic aerosol, the implement of ground-level ozone and so on [4,5,6,7]. Moreover, some VOCs are mutagenic, teratogenic, and carcinogenic for humans [8,9,10].

For this reason, several abatement techniques have been studied during the last years [11,12,13,14,15,16,17,18,19,20] and, among them, the heterogeneous photocatalysis is effective for the degradation of VOCs. Thus, many photocatalysts (mainly, TiO_2_-based materials) have been proposed for the VOC decomposition [21,22,23,24,25,26]. Specifically, titanium dioxide has created much interest and it is still currently used, both as such and doped with elements, for several photocatalytic applications. Moreover, the great interest in titania-based systems is due to the fact that TiO_2_ is a photoactive material at room temperature, has low toxicity, and exhibits various morphologies depending on the synthesis conditions [27,28].

However, pure TiO_2_ has photoactivity in the UV domain and this is a limitation for the possible use of this material in practical applications [29]. In order to widen the use of such material in the visible range, TiO_2_ is doped with either metals or nonmetals [30,31,32,33,34,35,36,37].

Several studies on titania-based catalysts have confirmed that the presence of dopants in the TiO_2_ framework may prevent the electron-hole recombination phenomena [38,39,40] and modify the absorption range in the UV-Vis spectra [41,42]. In addition, the insertion of such dopants in the TiO_2_ solid (e.g., P, Zr) significantly changes the physical properties of pure titania (e.g., surface area or crystallite size) which are very important parameters that play a key role in the activity [43,44].

The present work aims to study TiO_2_-based photocatalysts doped with phosphorus and zirconium. Specifically, a set of mesoporous samples was synthesized by a direct synthesis: TiO_2__A, TiO_2__B (titania prepared by two different procedures), P-TiO_2_ and Zr-TiO_2_ (binary oxides doped with either a nonmetal or metal). Then, the photocatalysts were tested for the total oxidation of propylene and ethylene as probe molecules of VOCs that are emitted by various sources (e.g., transportation, industries, and household activities) [11]. Complementary characterizations were done to investigate the physicochemical properties of the prepared samples, such as the N_2_ physisorption at 77 K, field emission scanning electron microscopy (FESEM), energy dispersive X-ray (EDX) analysis, XPS, and (DR)UV-Vis spectroscopy.

## 2. Materials and Methods

### 2.1. Catalysts Preparation: Pure Titania Samples

The sol-gel method was used to prepare the titania-based catalysts [11,45,46].

Briefly, a solution of 10 g of titanium (IV) butoxide (Sigma-Aldrich) and acetic acid (60 mL, 20 vol%, Sigma-Aldrich) was prepared dropwise in titanium (IV) butoxide and this mixture (titanium butoxide and acetic acid) was stirred at room temperature for 4 h. Meanwhile, 6 g of Pluronic 123 (Sigma-Aldrich) were dissolved in 40 mL of ethanol (Sigma-Aldrich) and stirred at room temperature for 4 h. Then, the solution with Pluronic 123 and ethanol was added dropwise to the first one prepared (titanium butoxide + acetic acid) and then stirred at room temperature for 24 h. The gel obtained was transferred in a Teflon autoclave and treated in the oven at 85 °C for 48 h. The precipitate was separated from the solution and dried overnight at 80 °C. The powder was calcined at 450 °C for 4 h with a heating rate of 1.8 °C·min^−1^. The pure TiO_2_ synthesized according to this procedure is denoted herein as “TiO_2__A”.

For comparison purposes, an additional TiO_2_ sample was also synthesized, using a method reported in [45]:

A solution of 1 mL of deionized H_2_O and 40 mL of anhydrous ethanol (Sigma-Aldrich, Germany was prepared. Subsequently, 1 mL of 12 M HCl (Sigma-Aldrich) solution was mixed with 12 mL of titanium (IV) butoxide (Sigma-Aldrich). Then, the solution with HCl and titanium butoxide was added dropwise into the previous one (solution of ethanol in water) and the mixture was subsequently stirred at room temperature for 24 h. The precipitate was separated, rinsed with deionized water, and dried overnight at 100 °C. The powder was calcined at 450 °C for 6 h with a heating rate of 1.8 °C·min^−1^ The sample is referred to herein as “TiO_2__B”.

In addition to the prepared catalysts, a commercial titania sample (P-25, Degussa), was included in the investigation as a reference material.

### 2.2. Catalysts Preparation: Doped Titania Samples

Doped titania samples were also synthesized to obtain new materials that enhance the photocatalytic activity. In the present work, the doping elements chosen were either phosphorus or zirconium [47].

The preparation of TiO_2_ doped with zirconium, denoted herein as Zr-TiO_2_, follows the same procedure of the synthesis of TiO_2__B (Section 2.1). However, it differs in the preparation of the solution of ethanol in water, where 2.27 g of ZrOCl_2_∙8H_2_O (Sigma-Aldrich) were dissolved. The Zr/Ti atomic ratio was expected to be about 0.2.

On the other hand, the preparation of the TiO_2_ sample doped with phosphorus, denoted herein as P-TiO_2_, follows the same procedure as the synthesis of TiO_2__A (Section 2.1) [46]. However, it differs in the preparation of the mixture of titanium butoxide and acetic acid, where 0.169 g of phosphoric acid (Sigma-Aldrich, 85% *w*/*w*) were added. The P/Ti atomic ratio was expected to be ~0.05.

### 2.3. Catalyst Characterization

The powder X-ray diffraction patterns were collected on an X’Pert Philips PW3040 diffractometer using Cu Ka radiation (2θ range = 10–70, step = 0.05°, time per step = 0.2 s). The diffraction peaks were indexed according to the Powder Data File database (PDF-2 1999, International Centre of Diffraction Data, Newton Square, PA, USA).

The specific surface area (S_BET_) and total pore volume (V_p_) were measured by means of the N_2_ physisorption at 77 K (Micromeritics Tristar II 3020, v1.03, Micromeritics Instrument Corp., Norcross, GA, USA, 2009) on samples previously outgassed at 200 °C for 4 h. The specific surface area of the samples was calculated using the Brunauer–Emmett–Teller (BET) method. The pore volume and pore diameter were estimated by the Barrett-Joyner-Halenda (BJH) method, during the desorption phase and the crystallite size was calculated by Scherrer’s equation.

The morphology of the samples was investigated by means of field emission scanning electron microscopy (FESEM Zeiss MERLIN, Gemini-II column, Oberkochen, Germany). Elemental analysis was carried out via energy dispersive X-ray (EDX) analysis (AZTec, Oxford Instruments, Abingdon, UK).

The chemical composition on the catalyst surfaces was investigated via X-ray photoelectron spectroscopy, performed in a PHI Versa probe apparatus using a band-pass energy of 187.85 eV, a 45° take-off angle, and 100 μm diameter X-ray spot size.

The samples were analyzed via the (DR)UV-Vis spectroscopy. This measurement was carried out through a UV-Vis double beam spectrophotometer (Varian Cary 500, Varian, Inc., Palo Alto, CA, USA). Spectralon^®^ was chosen as the reference for the background. The spectra were collected in 200–600 nm regions, with a resolution of 2 nm. The band gap energy was evaluated using the following equation [48]:

E (eV) = hc/λ(1)

where, hc is 1239.95 (eV nm) and λ (nm) is the absorption edge wavelength of the catalyst.

### 2.4. Photocatalytic Tests Under UV-Vis Light

Figure S1 shows the schematic diagram of the apparatus used for the photocatalytic tests. The apparatus was mainly comprised of a UV-Vis lamp (Newport Oriel Apex Illuminator which has a mixture of light of UVA in the range of 320–400 nm, UVB in the range of 290–320 nm and a portion of visible light in the range of 400–1050 nm, Milan, Italy) and a Pyrex reactor, where 0.5 g of catalyst powder was well dispersed. During the reaction, a mixture of 500 ppm-vol of VOCs (propylene or ethylene) and 10 vol% of O_2_ was continuously fed to the reactor and the products were analyzed using a non-dispersive infrared analyzer (NDIR, Hartmann-Braun, Milan, Italy) and a gas chromatograph (GC, Varian CP-3800, ShinCarbon ST column, FID, Milan, Italy). The distance between the UV-Vis lamp and the surface of the catalyst powder was fixed in such a way that the visible-light irradiance was maintained at 1000 W m^−2^. In a typical test, the gas feed was initially flowed through the reactor under dark condition to ”saturate” the catalyst with the probe molecule. When the system reached a steady-state condition (stable GC peak intensity), the lamp was turned on and the test ran for a time-on-stream of 3 h. The procedure of the data elaboration about these tests is described in the Supplementary Information.

## 3. Results and Discussion

### 3.1. X-Ray Diffraction (XRD) Analysis and N_2_ Physisorption at 77 K

**Figure 1:**
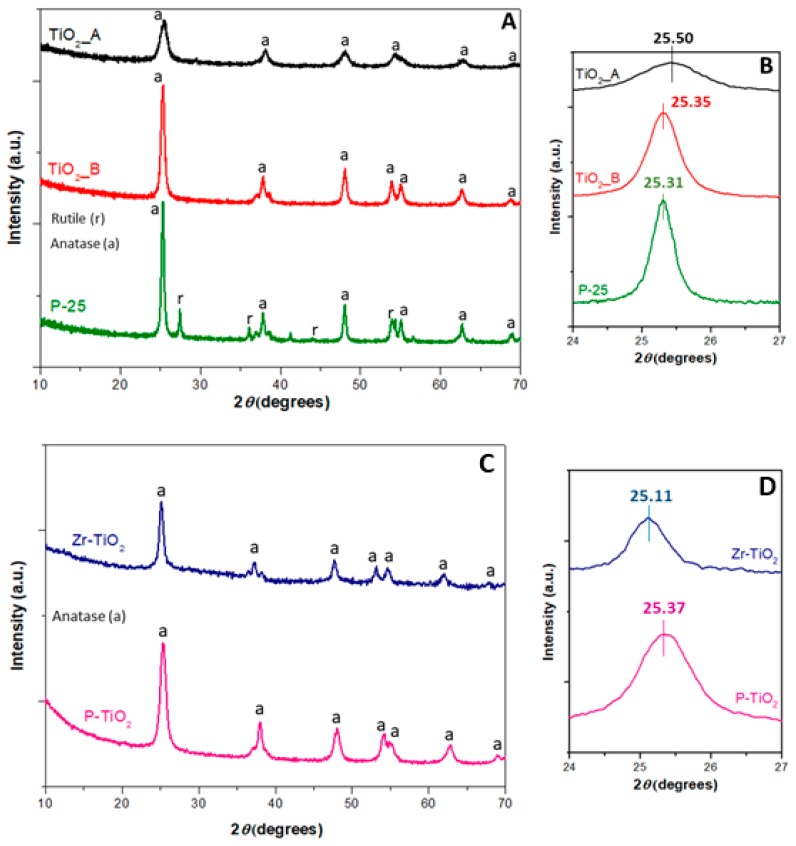
XRD patterns of pure and doped titania samples (**A** and **C**, respectively) and the magnifications of the main peaks (**B** and **D**, respectively).

Figure 1A shows the XRD spectra of TiO_2__A, TiO_2__B, and the P-25 samples, all featuring the diffraction peaks of anatase (a). No additional peaks are seen, therefore, indicating (a) the only presence of anatase. Converseley, the XRD pattern of P-25 shows the presence of both anatase and rutile (70:30 anatase to rutile ratio).

As is known, the anatase phase is more effective than the rutile in terms of photocatalytic activity, due to several textural and structural factors. In particular, the electron migration from the bulk to the surface in the anatase phase is faster than in the rutile phase, and for this reason, the recombination rate is lower [49]. However, the anatase-rutile mixed phase may provide a synergistic effect towards several photocatalytic reactions, especially when the two phases stay in close contact.

Moreover, the presence of both rutile and anatase phases enhances the photocatalytic activity since the intimate contact may favour the electron migration from rutile (conduction band) to anatase (trapping sites below the conduction band of rutile). This effect reduces the recombination rate in the rutile [49,50,51].

Figure 1B shows the magnification of the main peak in the spectra in the 2*θ* range of 24–27°. It is observed that the peaks in the spectra of TiO_2__A and TiO_2__B are shifted to a relatively higher 2*θ* with respect to that in the spectrum of P-25. Such shifts may reflect the structural differences among the samples (in terms of cell parameter). For example, it is possible to observe a general trend of the 2*θ* shift with the crystallite size calculated by the Scherrer’s equation (D_c_), as shown in Table 1. As a whole, it appears that the smaller D_c_ values the lower the 2*θ* values.

Figure 1C summarizes the XRD spectra of the doped titania samples. The lattice is characterized by only the presence of anatase. Moreover, no segregated phases (i.e., zirconium or phosphorus oxides) were observed in the XRD spectra of these samples, thus suggesting that the dopants are effectively dispersed and incorporated in the solid framework. Figure 1D shows the magnification of the main peak in the 2*θ* range of 24–27°. In the spectrum of Zr-TiO_2_ (blue curve), the peak seems shifted to a lower 2*θ* (signal at 25.11°) with respect to the peak position of TiO_2__B (signal at 25.35°). This shift may originate from the incorporation of the dopant in the titania structure. On the other hand, in the P-TiO_2_ spectrum the peak is shifted to a lower 2*θ* with respect to that of TiO_2__A.

The dopants into the titania framework may lead to different cell volumes and lattice parameters. We observed that TiO_2_ doped systems exhibit different trends as compared with those of the TiO_2_ samples. Specifically, the ionic radii of Zr^4+^ and Ti^4+^ cations are about 72 pm and 75 pm, respectively, thus explaining the easy metal substitution in the lattice and the similar crystallite sizes. On the other hand, for the P-TiO_2_ sample, it is possible to observe a change in the crystallite size (smaller values) if the phosphorus ions (52 pm) are introduced to the TiO_2_ lattice. Table 1 reports the crystallite sizes (*D*_C_) for the samples, as calculated by the Scherrer’s equation.

As a whole, particles with lower Dc values have a higher probability to have aboundant edges and corners on the solid surface as compared with the larger ones, for instance, that have higher amounts of terrace sites [52]. Due to the presence of these surface defects (i.e., edges and corners), the electron valence band shifts toward higher values because the local electron valence band on the surface increases. This phenomenon, along with the co-presence of the instauration, allows the absorption energy of atoms or molecules on the catalyt surface to increase. This means a major affinity between the catalyst surface and the molecules [53].

Table 1 also reports the results derived from the N_2_ physisorption at 77 K. The BET surface areas for the samples show an increasing trend according to the following order:

TiO_2__B (48 m^2^g^−1^) ≈ P-25 < TiO_2__A < Zr-TiO_2_ < P-TiO_2_ (153 m^2^g^−1^) (2)

It appears that smaller crystallites lead to higher S_BET_ values (e.g., P-TiO_2_). The two pure titania samples (TiO_2__A and TiO_2__B) have different S_BET_ values and this is possibly due to the different synthesis procedures. During the synthesis of the TiO_2__A, the hydrothermal treatment in the autoclave was carried out for 48 h. This aging step is crucial for the growth of meso-structures in the sample. In fact, this material is characterized by high porosity and surface area. Conversely, the synthesis of the TiO_2__B does not include the aging step and then worse textural properties occur

### 3.2. FESEM and Energy Dispersive X-Ray Analysis (EDX)

**Figure 2:**
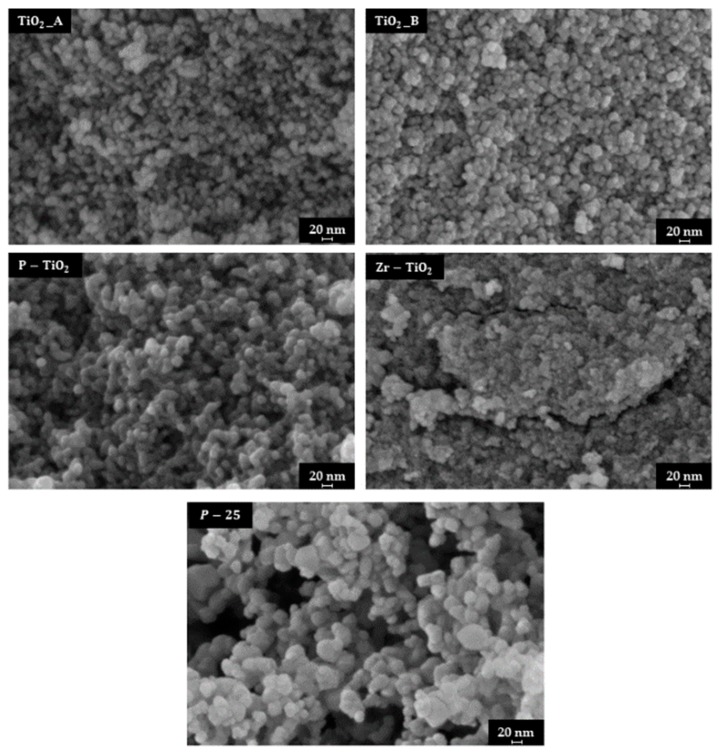
Field emission scanning electron microscopy (FESEM) micrograph of the sample studied.

Figure 2 shows the FESEM images of the prepared samples. The two pure titania samples exhibit the presence of nanoparticles (in the range 10–20 nm) agglomerated in clusters. On the other hand, the specific surface areas for these materials depend on both their textural and structural properties, as well the formation of the interparticle cavities (self-assembled NPs) may have a role on the SSA. Conversely, the XRD analysis (Scherrer’s equation) confirms the presence of different crystallite sizes for the prepared materials (see Table 1), specifically, it appears that the lower the crystallites size the higher the SSA.

The P-TiO_2_ sample is comprised of larger particles as compared with pure titania-based catalysts, whereas, the Zr-TiO_2_ has the most compact structure among the synthesized samples. For comparison purposes, the image of the P-25 sample was also added in Figure 2. In this case, this material is comprised of larger particles as compared with the others.

**Figure 3:**
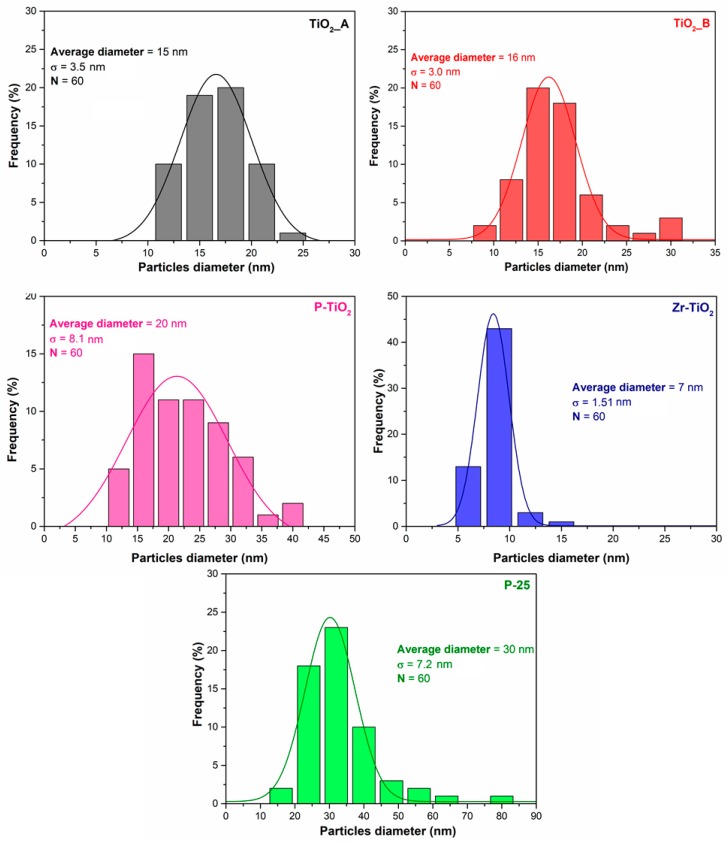
Particle size distribution of the samples studied.

The distribution of the particle sizes was carried out by using the ImageJ software [54]. For each sample, 60 particles were considered. Figure 3 reports the histogram of the particle size distribution, as well as the average particle diameter and the deviation standard (σ).

The average diameters of the TiO_2__A and of TiO_2__B samples are about 15 nm and 16 nm, respectively. The particle size distribution for the two samples is also comparable. Despite the resemblance of the morphology, the two samples have different surface areas (Section 3.1), with TiO_2__B having the lowest surface area. This means that TiO_2__A is comprised of larger pores inside the structure and rather than those between the particles (interparticle porosity).

The P-TiO_2_ sample is comprised of larger particles, with an average diameter of about 20 nm. This means that when P is incorporated in the TiO_2_ structure there is the formation of larger particles.

Smaller particles are observed with the Zr-TiO_2_ sample (approximately 7 nm). In fact, this sample exhibits a compact structure, as observed by FESEM. Despite its compactness, it exhibits a high surface area (136 m^2^·g^−1^). It is likely that the sample possess a high degree of intraparticle porosity, which crucially contributes to the overall porosity of the catalyst.

The P-25 sample shows the largest average diameter in the set of samples (about 30 nm). Nevertheless, this sample has the lowest surface area. Therefore, it appears that P-25 have a lower intraparticle porosity as compared with, for example, with Zr-TiO_2_.

Table 2 summarizes the elemental composition of the doped titania samples derived from the EDX analysis. The atomic percentage of O is 70% for both samples. The percentage of Ti is 28% for P-TiO_2_ and 26% for Zr-TiO_2_. Through these values, it is possible to evaluate the relative abundance of P and Zr to verify the dopant-to-Ti ratio (P/Ti and Zr/Ti). It was found that the P/Ti is about 5 at%, while the Zr/Ti is close to 18 at%. This confirms the theoretical dopant loading expected prior to the synthesis.

**Figure 4:**
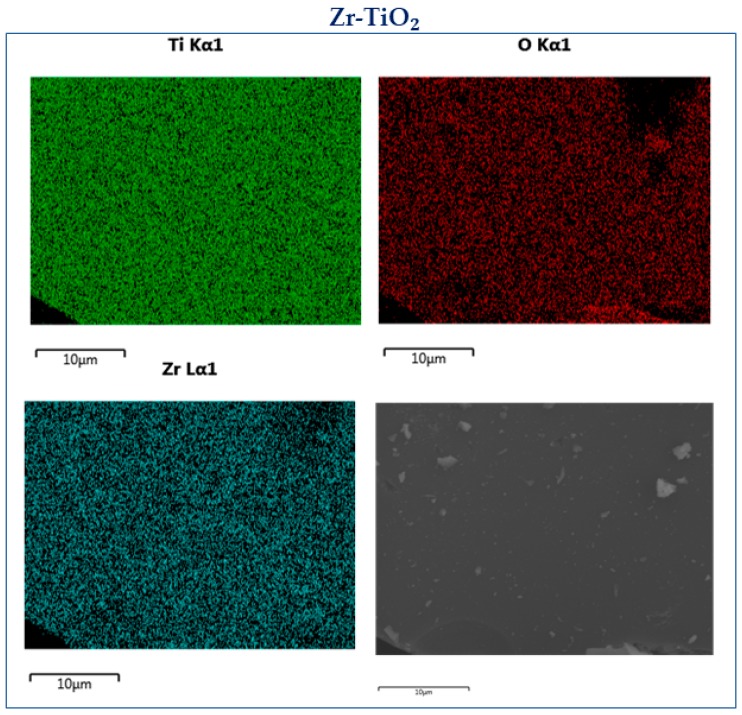
EDX maps over the Zr-doped sample.

**Figure 5:**
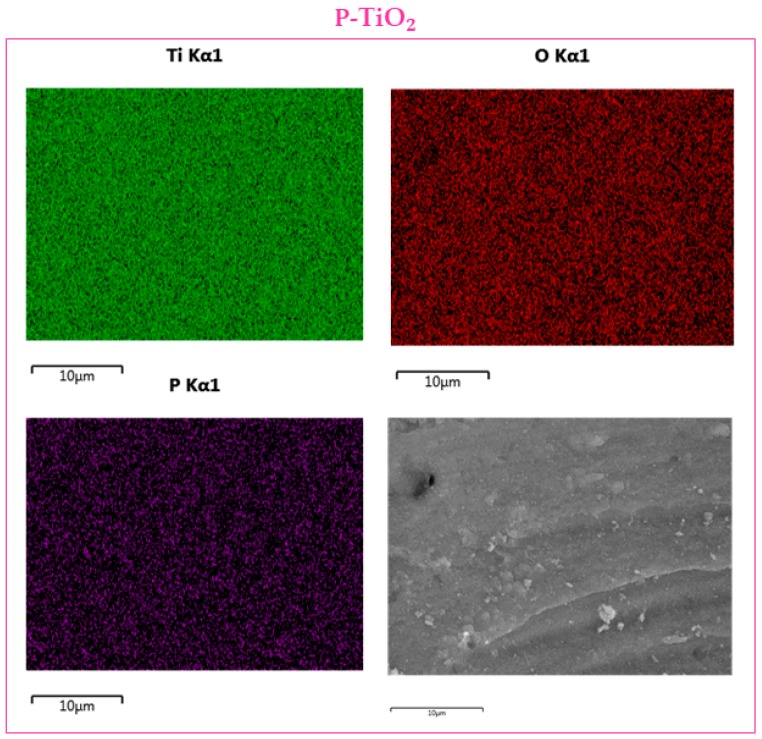
EDX maps over the P-doped sample.

Figure 4 and Figure 5 report the EDX maps for the doped samples. The elemental mapping is a useful method to analyze the distribution of the elements in the sample. Both dopants, Zr (Figure 4, light blue) and P (Figure 5, violet) seem to be effectively distributed in their respective samples. This indicates the benefit of using the adopted techniques for maintaining a good dopant distribution in the samples.

### 3.3. (DR)UV-Vis Spectroscopy

**Figure 6:**
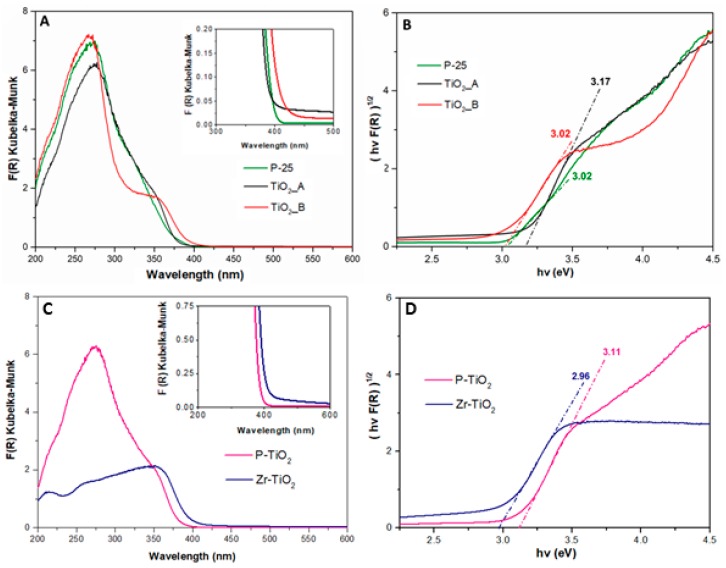
(DR)UV-Vis spectra of the studied sample: pure titania samples ((**A**) Kubelka≠Munk plot and (**B**) Tauc’s plot) and doped samples ((**C**) Kubelka–Munk plot and (**D**) Tauc’s plot).

Figure 6 reports the (DR)UV-Vis spectra of the studied samples.

Compared to P-25, the TiO_2__A and TiO_2__B samples absorb radiation in a broader range of wavelength (i.e., this absorption occurs at 425 and 350 nm, as shown by the inset on Figure 6A). The absorption by the TiO_2__A sample is observed at 375 and 330 nm, due to charge transfer (CT) transitions in the solid.

Figure 6B shows the corresponding Tauc’s plot, used to evaluate the band gap energies of the samples which are summarized in Table 3 with their absorption edge wavelength. The values of λ are also reported in Table 3.

Among the pure titania samples, TiO_2__A has the highest value of band gap energy (approximately 3.17 eV), while Zr-TiO_2_ has the lowest value, 2.96 eV. From the point of view of band gap energy, the most potentially active catalyst seems to be Zr-TiO_2_ because the energy required to promote electrons from the valence to conduction band is the lowest.

Similarly, Figure 6C shows the (DR)UV-Vis spectra of the doped titania samples. The Zr-TiO_2_ sample has a range of absorption in the visible light region, as evidenced by the inset in Figure 6C. Figure 6D shows the corresponding Tauc’s plot of the spectra and the band gap energy evaluated (these values are reported in Table 3 with the absorption wavelength edge of the samples).

### 3.4. X-ray Photoelectron Spectroscopy (XPS)

Table 4 shows the surface atomic percentage of the elements on the catalysts analyzed via XPS. In most cases, the Ti-to-O ratio is about one to three, lower than the theoretical ratio for a pure TiO_2_ (one to two). This means that the catalysts have a higher oxygen abundance on the surface than in the bulk. The Ti-to-O ratio for Zr-TiO_2_ seems lower than the average value, nevertheless, the slightly lower Ti quantity is actually compensated by a small percentage of Zr.

**Figure 7:**
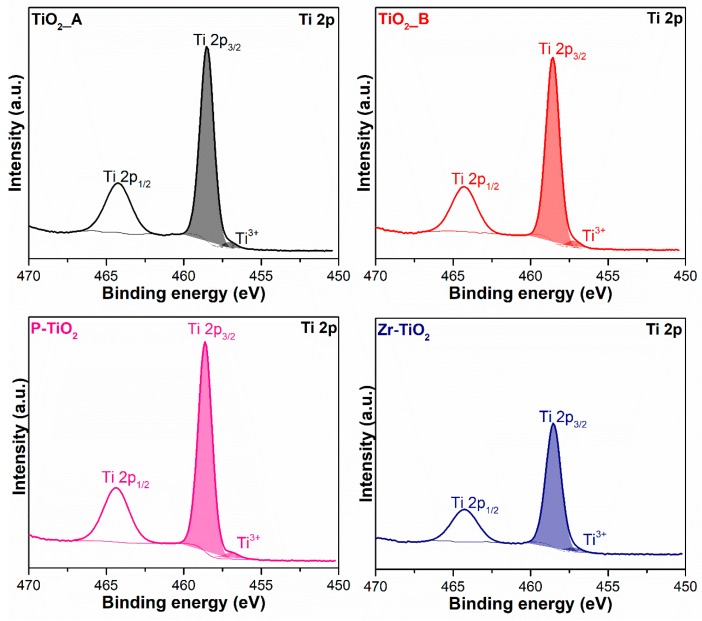
X-ray photoelectron spectra of the samples in the Ti 2*p* core level.

Figure 7 shows the Ti 2*p* XP spectra of all the samples, which demonstrate a high-intensity spin-orbit peak in the 2*p*_3/2_ region (average binding energy (BE) = 458.6 eV) and a low-intensity peak in the 2*p*_1/2_ region, with a spin orbit splitting (Δ) of about 5.7 eV. All the spectra of the samples, regardless of the dopant, demonstrate a typical Ti 2*p* spectrum of Ti^4+^ in TiO_2_ [55]. Two peak components have been deconvoluted in the Ti 2*p*_3/2_ region. The former centered at about 458.6 eV is ascribed to Ti^4+^ while the latter centered at around 457.1 eV is assigned to Ti^3+^ [55]. Table 5 summarizes the relative abundances of Ti species on the surface of the catalysts. In general, the abundance of Ti^3+^ on the surface is much lower than that of Ti^4+^. Apparently, Zr-TiO_2_ has the highest Ti^3+^ abundance among the samples and this is most likely caused by the weakening of Ti–O–Ti bond induced by cation substitution with Zr, which has a larger ionic radius (0.75 and 0.61 Å for Zr^4+^ and Ti^4+^, respectively) [55].

**Figure 8:**
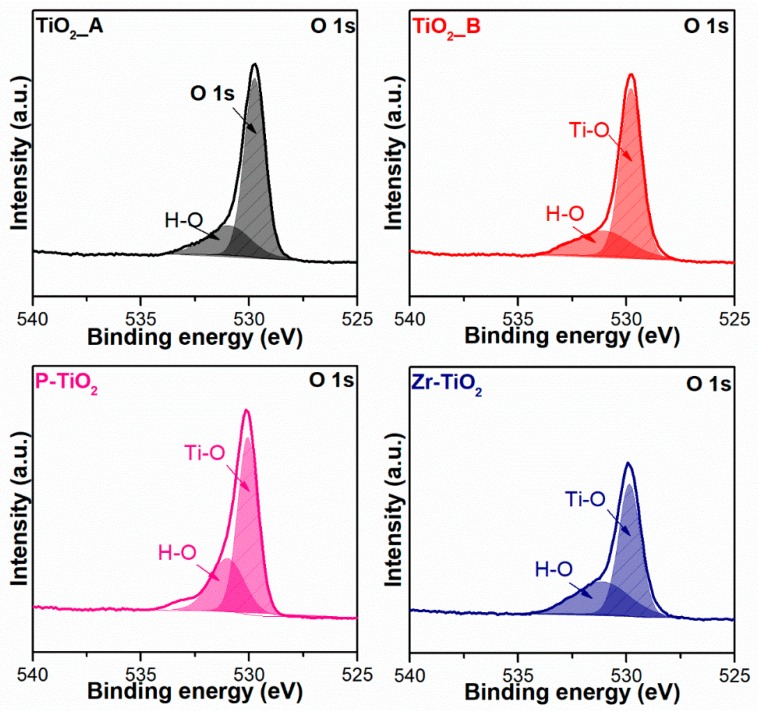
Deconvoluted X-ray photoelectron spectra of the samples in the O 1*s* core level.

Figure 8 shows the O 1*s* XP spectra of the samples. In most cases, two peaks corresponding to the Ti-O (lattice-like oxygen species bonded with Ti) and the non-lattice oxygen (NLO) species, related to defect sites such as hydroxyls (O–H), were identified at 531 eV and 529 eV, respectively [56,57,58]. In the case of P-TiO_2_, two NLO species were identified at 530.9 eV and 533.1 eV and they were associated with oxygen from phosphate and hydroxyls, respectively [55].

Table 6 reports the relative abundances of both species evaluated through the curve fitting of the XP spectra. It is worth noting that the doped samples have a higher abundance of NLO species on the surface (38% for Zr-doped and 36% for P-doped sample) than the pure titania ones. The higher abundance of such species, such as OH groups on the surface, may bestow on the doped samples an improved ability to mediate VOC abatement. This enhancement may be because the hydroxyl groups on the surface are transformed via oxidation to the OH radicals, which may participate in the photocatalytic VOC oxidation [59].

**Figure 9:**
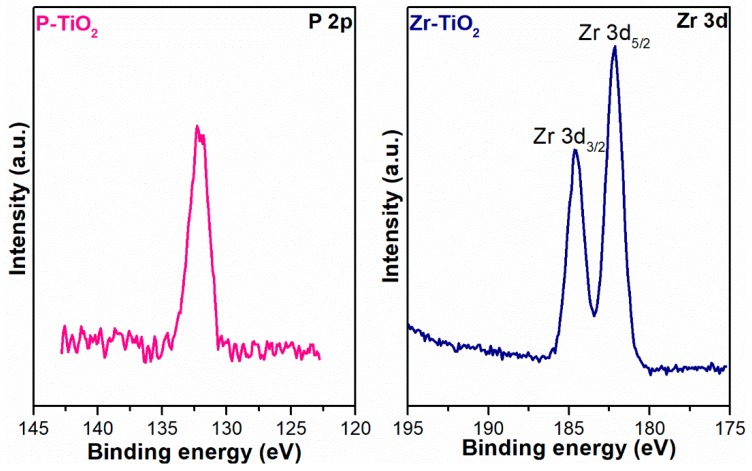
X-ray photoelectron spectra of P 2*p* and Zr 3*d* of P-TiO_2_ and Zr-TiO_2_, respectively.

Figure 9 shows the P 2*p* and Zr 3*d* XP spectra of P-TiO_2_ and Zr-TiO_2_, respectively. The relative abundances of these dopants with respect to the constituting elements of the parent oxide (i.e., Ti and O) are reported in Table 4. The percentage of P on the surface of the P-TiO_2_ sample is about 3 at%, while the percentage of Zr on the surface of Zr-TiO_2_ sample is around 4 at%, as shown in Table 4. From the XPS analysis, the P-TiO_2_ sample has about 3 at% of P on the surface, while from the EDX analysis the sample was observed to have about 2 at% of P in the bulk. This means that the dopant is more concentrated on the TiO_2_ surface than in the bulk. Zr has the same atomic percentage in the bulk of Zr-TiO_2_ as well as on the surface. The P 2*p* spectrum of P-TiO_2_ sample shows a peak at 132.3 eV, which corresponds to phosphate bonded to Ti [55].

The spin orbit peak of Zr 3*d* in the 3*d*_5/2_ region is centered at 182.2 eV, which is simply ascribed to Zr^4+^ [55]. In the pure titania samples, there are no differences in the titanium and oxygen compositions, which are 25 at% and 75 at%, respectively, in all cases, as shown in Table 4. When the dopant was inserted in the TiO_2_ framework, the abundance of titanium and oxygen changes. The titanium abundance in P-TiO_2_ is the same as that in pure TiO_2_, while the oxygen abundance decreases from 75 at% to 72 at%. In Zr-TiO_2_ the titanium abundance increases from 75 at% to 77 at%, while the oxygen abundance decreases from 25 at% to 19 at%.

### 3.5. Photocatalytic Activity

In the photocatalytic reaction, the electrons in the TiO_2_ structure are promoted to the anatase conduction band (CB) and the corresponding holes are created in the valence band (VB). During this phenomenon, the ·OH and ·O_2_^−^ species are produced, according to the following Equations (1)–(4) [60]:

TiO_2_ +*hν*→ *h*^+^(VB) + e^−^(CB) (3)

H_2_O + *h*^+^(VB) → OH^−^ + H^+^ (4)

*h*^+^(VB) + OH^−^→ ·OH (5)

O_2_ + e^−^(CB) → ·O_2_^−^ (6)

These radical species additionally react with ethylene (5–8) to produce CO_2_ and water vapor, represented in the following equations:

·OH + C_2_H_4_→ (C_2_H_4_)∗ (7)

(C_2_H_4_OH)∗ +·O_2_→ CO_2_ +H_2_O (8)

Ti(IV) + e^−^→ Ti(III) (9)

Ti(III) + O_2_→ Ti(IV) + O_2_^−^ (10)

A similar reaction mechanism is observed for the photocatalytic oxidation of propylene (9–12):

·OH + C_3_H_5_→ (C_3_H_5_OH)∗ (11)

(C_3_H_5_OH)∗ + ·O_2_→ CO_2_ +H_2_O (12)

Ti(IV) + e^−^→ Ti(III) (13)

Ti(III) + O_2_→ Ti(IV) + O_2_^−^ (14)

**Figure 10:**
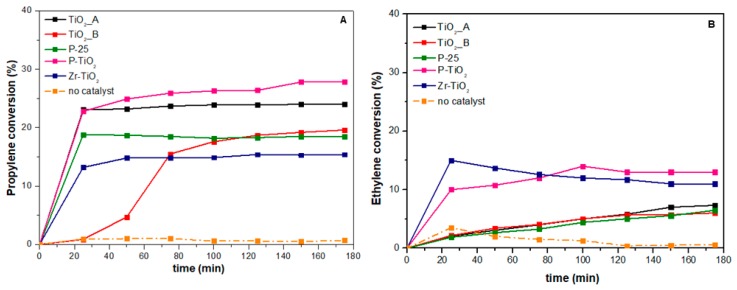
Trends of the propylene (**A**) and ethylene (**B**) conversions (%) over the time-on-stream.

Figure 10 shows the conversion of propylene (10A) and ethylene (10B) achieved during the photocatalytic tests. For comparison purposes, the conversion of the uncatalyzed reaction has also been included in Figure 10 and it is represented by the orange curve. Table 7 summarizes the VOC conversion values for all the catalysts.

As a whole, the catalytic performances (in terms of conversion) of the prepared samples is summarized as follows:

Propylene: Zr-TiO_2_ (15.4%) < P-25 < TiO_2__B < TiO_2__A < P-TiO_2_ (27.8%)

Ethylene: TiO_2__B (6%) < P-25 < TiO_2__A < Zr-TiO_2_ < P-TiO_2_ (13%)

The most performing catalyst for both oxidation reactions appears to be the P-TiO_2_ (evaluated with TOS = 3 h). In fact, the propylene conversion achieved over this catalyst was about 27.8%, while the conversion of ethylene was close to 13%.

In the case of propylene oxidation (Figure 10A), the two meso-structured samples (P-TiO_2_ and TiO_2__A) seem to give a higher catalytic activity towards the reaction than the other samples. This suggests that the propylene oxidation reaction is textural dependent on these catalysts. The activity of the reaction is affected by the catalyst structure and morphology. On the basis of the previous N_2_ physisorption and FESEM analyses, the meso-structure in the two samples brings about high surface area and large pore size. Propylene is a bulkier molecule than ethylene. Therefore, it is easily adsorbed on the catalyst surface, the diffusion limitation must be minimized, and the meso-structure helps tackle such a diffusional barrier.

The commercial sample, P-25, despite being relatively less active than the two meso-structured samples, induced a fast light-off reaction. From Figure 10A, we observed that the conversion increased quickly to about 18% in the first few minutes. Although the sample is not morphologically endowed, the synergism between rutile and anatase might contribute to the initial catalytic activity.

The effect of dopant seems variable in the propylene oxidation. While phosphorus gives a beneficial effect on catalyst morphology and, subsequently, on catalytic activity, zirconium seems to disfavor this effect. This finding may be linked to its effect on morphology, as the incorporation of Zr, according to the N_2_ physisorption results (vide supra), leads to higher surface area but lower porosity. The contribution of surface area was appreciable in the first few minutes of the illumination, where the reaction ignited very quickly over Zr-TiO_2_ than over TiO_2_. However, the reaction proceeded more slowly afterwards as it was limited by the diffusion phenomena (due to smaller pore size).

In the case of ethylene oxidation (Figure 10B), the P-TiO_2_ and Zr-TiO_2_ catalysts exhibited the highest conversion, whereas, the titania samples showed lower but comparable performances. Despite being equally active, the two most performing catalysts differ in morphology. This means that the ethylene oxidation reaction is not textural dependent on these samples. Variations in catalyst morphology hardly affect the catalytic performance. This is reasonable because ethylene is a simpler molecule than propylene and its smaller size should not be constrained by diffusion limitation. However, it seems that the reaction is affected by the presence of dopants. These foreign elements are traditionally introduced to titania lattice to narrow the band gap energy and, subsequently, to expand the absorption spectrum. The variation in band gap energy among the samples, as summarized in Table 3, is, nevertheless, minor and UV irradiation is dominant. This means that another effect coming from the dopant plays a more important role in the catalysis and that could be the generation of defect sites on the surface. The XPS analysis (vide supra) has highlighted the increased quantity of OH groups, which are indicative of defect sites, on the surface of the doped titania samples.

Finally, a fairer comparison of catalytic activity among the catalysts was carried out by analyzing their specific reaction rates calculated at the end of the TOS (3 h). The rates were normalized with respect to catalyst weight (µmol h^−1^ g_cat_^−1^), as well as to specific surface area (µmol h^−1^ m^−2^). The latter specific rate eventually referred to the catalyst intrinsic activity.

**Figure 11:**
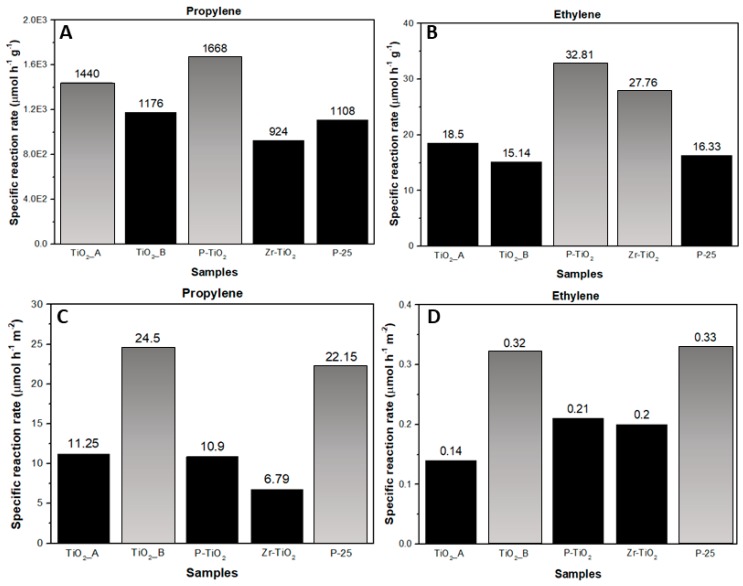
Specific reaction rates of the catalysts for the propylene (**A**,**C**) and ethylene (**B**,**D**) oxidation reactions.

Figure 11A,B summarize the values of the specific rates of propylene and ethylene oxidation for all catalysts normalized with respect to the catalyst weight. As we previously observed, the most active catalysts for propylene oxidation, according to their specific reaction rates, were P-TiO_2_ and TiO_2__A (specific reaction rates = 1668 and 1440 µmol h^−1^ g^−1^, respectively) and those for ethylene oxidation were P-TiO_2_ and Zr-TiO_2_ (32.81 and 27.76 µmol h^−1^ g^−1^). For propylene oxidation, the trend for the specific reaction rate is in accordance with the one observed for the specific surface area (153 m^2^ g^−1^ for P-TiO_2_ and 128 m^2^ g^−1^ for TiO_2__A, the highest in the series). The Zr-TiO_2_ sample has the lowest specific reaction rate (approximately 924 µmol h^−1^ g^−1^) despite its high surface area (136 m^2^ g^−1^). The reason could be the morphology of this sample, i.e., the small particle dimension and the compactness reduces the accessibility of propylene to the active sites. For ethylene oxidation, the two most active catalysts are among those that have the highest surface area. However, the activity seems to be more affected by the presence of dopants (i.e., P and Zr), which likely modifies the surface chemical and electronic properties of the catalysts and plays a key role in the reaction mechanism.

Finally, Figure 11C,D summarizes the values of the specific rates of propylene and ethylene oxidation for all catalysts normalized with respect to the catalyst surface area. The two low-surface area catalysts (TiO_2__B and P-25) were apparently the most intrinsically active catalysts for both oxidation reactions. From the XRD and FESEM micrography analysis, the two samples have the highest average particle diameters and average crystallite sizes (D_c_). Moreover, in the case of TiO_2__B and P-25, the average particle diameter is equal to the average D_c_. This means that the two samples are largely comprised of monocrystalline particles, which likely contain fewer bulk defects. It is widely believed that the presence of structural defects, regardless of their position in the catalyst (i.e., in the bulk or on the surface), affect the overall photocatalytic activity. Bulk defects are known to act as recombination centers for electron-hole pairs, whereas, surface defect sites act as traps for the photogenerated electrons [61,62,63,64]. While the former negatively impacts the photocatalytic activity, the latter may positively contribute to the photocatalytic activity as they extend the lifetime of holes. Fewer bulk defects in P-25 and TiO_2__B, due to the predominance of monocrystalline particles, may inhibit the electron-hole recombination. It is also known that for single crystal nanoparticles, smaller particles likely contain more edge and corner sites on the surface while larger particles contain more terrace sites (hence less surface defect sites) [46]. Since both P-25 and TiO_2__B are characterized by larger particles, it is surmised that the two samples have fewer surface defect sites, thus a lower possibility of electron trapping.

## 4. Conclusions

In this work, several titania-based catalysts were synthesized: two pure titania catalysts (TiO_2__A and TiO_2__B) and two doped titania catalysts (P-TiO_2_ and Zr-TiO_2_). As a whole, the presence of either P or Zr species improves the SSA and total pore volume of solids. Specifically, the P-TiO_2_ sample exhibits the best textural properties (S_BET_ = 153 m^2^g^−1^ V_p_ =0.74 cm^3^g^−1^). Both the XRD and EDX analyses confirmed the incorporation of either P or Zr into the TiO_2_ framework and no segregated phases (namely, Zr- or P-oxides) were revealed. The catalysts were tested for the propylene and ethylene oxidation under UV-Vis light. The P-TiO_2_ sample resulted in being the most effective photocatalyst for both the reactions (propylene conversion = 27.8% TOS = 3 h; ethylene conversion = 13% TOS = 3 h).

## Figures and Tables

**Table 1:** Textural and structural properties of the prepared samples.

Sample	S_BET_ (m^2^ g^−1^) ^a^	V_p_ (cm^3^ g^−1^) ^b^	D_p_ (nm) ^b^	D_c_ (nm) ^c^
TiO_2__A	128	0.34	12	14
TiO_2__B	48	0.10	5	16
P-TiO_2_	153	0.74	16	12
Zr-TiO_2_	136	0.13	4	15
P-25	50	0.19	13	30

^a^ Specific surface area calculated by the BET method; ^b^ pore volume and pore diameter estimated by the BJH method, during the desorption phase; ^c^ crystallite size calculated by the Scherrer’s equation.

**Table 2:** Energy dispersive X-ray (EDX) analysis over the doped samples.

Element	P-TiO_2_ (at%)	Zr-TiO_2_ (at%)
O	70	70
P	2	-
Zr	-	4
Ti	28	26
Total	100	100

All the atomic percentages are the average on three different areas.

**Table 3:** Band gap energy (eV) and the absorption edge wavelength (nm) of the synthesized samples.

Sample	Absorption Edge Wavelength, λ (nm)	Band Gap Energy, E (eV)
TiO_2__A	391	3.17
TiO_2__B	411	3.02
P-TiO_2_	399	3.11
Zr-TiO_2_	419	2.96
P-25	411	3.02

**Table 4:** Atomic percentage of the elements on the catalyst surfaces.

Catalyst	Atomic %			
	Ti	O	P	Zr
TiO_2__A	25	75	-	-
TiO_2__B	25	75	-	-
P-TiO_2_	25	72	3	-
Zr-TiO_2_	19	77	-	4
P25	27	73	-	-

**Table 5:** Relative abundances of Ti species (in atomic percentage) derived from deconvoluted Ti 2*p* XP spectra.

Catalyst	Ti 2p			
	Ti^3+^		Ti^4+^	
	(% atom)	BE (eV)	(% atom)	BE (eV)
TiO_2__A	3	457.0	97	458.5
TiO_2__B	5	457.2	95	458.6
P-TiO_2_	3	457.1	97	458.9
Zr-TiO_2_	9	457.2	91	458.5
P25	3	457.2	97	458.7

**Table 6:** Relative abundances of O species (in atomic percentage) derived from deconvoluted O 1*s* XP spectra.

Catalyst	O 1*s*					
	Ti-O		P-O		NLO *	
	(% atom)	BE (eV)	(% atom)	BE (eV)	(% atom)	BE (eV)
TiO_2__A	74	529.7	-	-	26	531.0
TiO_2__B	72	529.8	-	-	28	531.1
P-TiO_2_	64	530.0	32	530.9	4	533.1
Zr-TiO_2_	62	529.9	-	-	38	531.1
P25	85	529.9	-	-	15	531.3

* NLO = non-lattice oxygen.

**Table 7:** Conversions (%) of propylene and ethylene under UV-Vis light (TOS = 3 h).

Sample	Propylene Conversion (%)	Ethylene Conversion (%)
TiO_2__A	24.0	7.3
TiO_2__B	19.6	6.0
P-TiO_2_	27.8	13.0
Zr-TiO_2_	15.4	11.0
P-25	18.5	6.5

## Supplementary Materials

The following are available online at https://www.mdpi.com/1996-1944/12/13/2121/s1, Figure S1: Schematic diagram of the apparatus used for the photocatalytic tests.
