# Supplementary material for: Photocatalytic Abatement of Volatile Organic Compounds by TiO2 Nanoparticles Doped with Either Phosphorous or Zirconium
Source: Materials (Basel). 2019 Jul 1;12(13):2121. doi: 10.3390/ma12132121 (PMC6651007; doi:10.3390/ma12132121)
Supplement: Supplementary file 1 [file materials-12-02121-s001.pdf]

Supplementary Materials: Photocatalytic Abatement of Volatile Organic Compounds by TiO_2 Nanoparticles Doped with Either Phosphorous or Zirconium

Melodj Dosa, Marco Piumetti *, Samir Bensaid, Tahrizi Andana, Camilla Galletti, Debora Fino and Nunzio Russo

Department of Applied Science and Technology, Politecnico di Torino, Corso Duca degli Abruzzi 24, 10129 Turin, Italy

* Correspondence: marco.piumetti@polito.it;

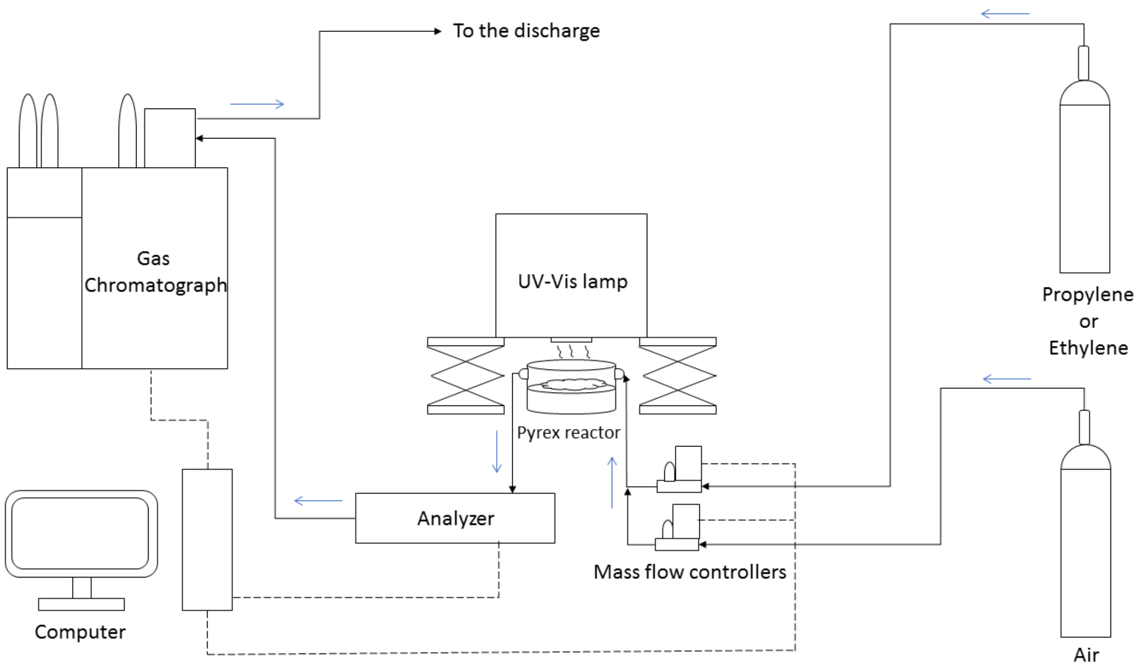

Figure S1. Schematic diagram of the apparatus used for the photocatalytic tests.

Procedure for the GC Data Elaboration

During the dark condition, the photocatalyst surface was saturated with either ethylene or propylene to reach a steady-state condition (the peak in the GC spectrum does not change). The peak area reflects the initial amount of VOC in the flow (500 ppmv of propylene or ethylene).

When the UV-Vis source was switched on, the area of the peak decreases over the time.

The conversion was calculated as the follow Equation (2):

$$Conversion\ (\%) = \frac{Peak\ area_{t=0} - Peak\ area_{t_i}}{Peak\ area_{t=0}} \quad (2)$$

where *Conversion (%)* is the propylene or ethylene converted at the time t_i; *Peak area*$_{t=0}$ is the peak area at time 0 (= dark condition) *Peak area*$_{ti}$ is the peak area at time i (from 0 to 3 h).
